# Effects of the rice-mushroom rotation pattern on soil properties and microbial community succession in paddy fields

**DOI:** 10.3389/fmicb.2024.1449922

**Published:** 2024-07-24

**Authors:** Haibo Hao, Yihong Yue, Qian Wang, Tingting Xiao, Zelong Zhao, Jinjing Zhang, Hui Chen

**Affiliations:** ^1^National Research Center for Edible Fungi Biotechnology and Engineering, Key Laboratory of Applied Mycological Resources and Utilization, Shanghai Key Laboratory of Agricultural Genetics and Breeding, Ministry of Agriculture, Institute of Edible Fungi, Shanghai Academy of Agricultural Sciences, Shanghai, China; ^2^State Key Laboratory of Genetic Engineering and Fudan Center for Genetic Diversity and Designing Agriculture, Institute of Plant Biology, School of Life Sciences, Fudan University, Shanghai, China; ^3^Shanghai Biozeron Biotechnology Co., Ltd., Shanghai, China

**Keywords:** *Stropharia rugosoannulata*, straw degradation, soil nutrients, microbial community, co-occurrence networks, structural equation model

## Abstract

**Introduction:**

Currently, straw biodegradation and soil improvement in rice-mushroom rotation systems have attracted much attention. However, there is still a lack of studies on the effects of rice-mushroom rotation on yield, soil properties and microbial succession.

**Methods:**

In this study, no treatment (CK), green manure return (GM) and rice straw return (RS) were used as controls to fully evaluate the effect of *Stropharia rugosoannulata* cultivation substrate return (SRS) on soil properties and microorganisms.

**Results:**

The results indicated that rice yield, soil nutrient (organic matter, organic carbon, total nitrogen, available nitrogen and available potassium) and soil enzyme (urease, saccharase, lignin peroxidase and laccase) activities had positive responses to the rice-mushroom rotation. At the interannual level, microbial diversity varied significantly among treatments, with the rice-mushroom rotation significantly increasing the relative alpha diversity index of soil bacteria and enriching beneficial microbial communities such as *Rhizobium*, *Bacillus* and *Trichoderma* for rice growth. Soil nutrients and enzymatic activities were significantly correlated with microbial communities during rice-mushroom rotation. The fungal-bacterial co-occurrence networks were modular, and Latescibacterota, Chloroflexi, Gemmatimonadota and Patescibacteria were closely related to the accumulation of nutrients in the soil. The structural equation model (SEM) showed that fungal diversity responded more to changes in soil nutrients than did bacterial diversity.

**Discussion:**

Overall, the rice-mushroom rotation model improved soil nutrients and rice yields, enriched beneficial microorganisms and maintained microbial diversity. This study provides new insights into the use of *S. rugosoannulata* cultivation substrates in the sustainable development of agroecosystems.

## Introduction

Ecological agriculture organically combines agriculture and ecology to promote the efficient, economical and sustainable development of agriculture (Jia et al., 2022; Wang et al., 2022). The continuous cultivation of staple food crops (e.g., rice) in traditional agriculture still faces problems such as land degradation, loss of soil fertility and outbreaks of plant diseases and pests (Singh et al., 2020; Lu et al., 2021). In recent years, the use of spent mushroom substrates from edible mushrooms has effectively improved the soil nutrients and ecological structure of paddy fields and saline-alkaline marshes (Chen et al., 2022; Li et al., 2023). In the rice-mushroom rotation system, mycelia decompose a large amount of straw, and the resulting organic matter is transferred to the soil to improve soil nutrients (Hu et al., 2023). Based on the application value of rice-mushroom rotation in ecological agriculture, it is very important to analyze its potential sustainable mechanism.

Crop rotation can enhance species diversity and promote ecosystem services, such as pest and disease control, carbon storage, and soil fertility (Beillouin et al., 2021). The advantage of crop rotation is that it reduces the input of external nutrients and stabilizes production (Zhao et al., 2022). Due to the high protein content of legume crops, they can significantly increase rice yield as auxiliary rotation plants, and the rice-green manure (broad bean) rotation system is widely promoted in China (Bai et al., 2022; Zhao et al., 2022). Moreover, rice-green manure rotation increased soil organic matter (SOM), humic acid (HA), total N, and soil alkaline phosphatase, which were the main reasons for the increase in rice yield (Bai et al., 2022). In addition, crop rotation systems such as rice-wheat, rice-potato with rice straw mulch and rice-oilseed rape also increase rice yields (Chen et al., 2018). Therefore, crop rotation improves soil properties and thus increases rice yield. However, the effect of rice-mushroom rotation on rice yield has not been reported, and this crop rotation pattern has not been systematically studied in ecological agriculture.

At present, straw return is still an important way to increase the storage of organic carbon in farmland (Liu et al., 2014). The advantages of returning straw to the field are that the soil fertility, macroaggregates and porosity improve after straw decomposition, which is beneficial to rice yield (Liu et al., 2014). However, its limitation lies in the fact that many straw types are difficult to degrade under natural conditions, and straw types that are not fully degraded easily cause disease and reduce rice yield (Song et al., 2023). Therefore, the selection of suitable ascomycetes (*Trichoderma viride*, *Penicillium*, and *Phanerochaete chrysosporium*) or basidiomycetes (*Agaricus bisporus* and *Stropharia rugosoannulata*) to accelerate the transformation of straw will be highly beneficial for promoting agricultural cycles and increasing crop yields (Chen et al., 2022; Hu et al., 2023).

Soil microorganisms contribute mainly to maintaining the multifunctionality of ecosystems and promoting changes in soil physical and chemical properties (Jiao et al., 2021). Most crop rotations to improve soil nutrients are regulated by soil microorganisms through complex biochemical reactions (Liu et al., 2020). Many crop rotation experiments have demonstrated that crop rotation can increase soil microbial abundance (Zuber et al., 2018), change soil enzyme activity (Jiao and Yuan, 2019) and affect microbial composition (Liu et al., 2020). For example, compared with rice-fallow manure, rice-wheat and rice-green manure significantly increase microbial abundance and diversity (Feng et al., 2020). In addition, soil microorganisms can respond positively to mushroom residues returned to the soil. Therefore, studying the succession of soil microbial communities will play an important role in ecological agriculture.

This study mainly addressed three objectives: (1) To comprehensively evaluate the effects of long-term SRS return on soil physicochemical indices and rice yield based on the use of conventional green manure return and straw return as references. (2) To assess the differences in soil microbial diversity and composition between the SRS return treatment and the other treatments at the interannual level. (3) To explore the relationship between the physicochemical properties of long-term SRS-returned soil and the succession of microbial communities. We hypothesized that soil physicochemical properties, soil microorganisms and rice yield respond positively to the return of SRS to the field, and these effects are related to nutrient accumulation caused by the transfer of cultivation substrates of edible fungi to the soil during the rice-mushroom rotation.

## Materials and methods

### Site selection and experimental description

#### Experimental design

The experimental site was selected in Jianshe town (31°38′N, 121°28′E), Chongming District, Shanghai city, which is one of the main rice-producing areas of Shanghai. The rice variety cultivated in rotation was Nanjing 46. The basic soil properties were as follows: organic matter 2.37%, organic carbon 1.36%, total nitrogen 1.02%, available nitrogen 40.45 mg/kg, total potassium 9.57 g/kg, available potassium 29.42 mg/kg, total phosphorus 0.39 g/kg, available phosphorus 23.19 mg/kg, water-soluble total salt 1.49 g/kg, and pH 7.46. The experiment included four treatments: a control (CK), GM, straw returned to the field (RS) and the cultivation substrate of *S. rugosoannulata* returned to the field (SRS). The scenarios of rice rotation with different treatments are shown in Supplementary Figure S1.

#### Experimental details

The area of the paddy field for each treatment was approximately 30 × 40 m (length × width). In the first year, various experimental treatments were carried out after the rice harvest in October 2019. CK: control, i.e., no treatment. GM: Green manure (faba bean) was generally sown in November, and the soil was plowed with a rotary cultivator in April of the second year. RS: The soil was plowed with a rotary cultivator after the straw was returned to the field. SRS: *S. rugosoannulata* was utilized to degrade 30 kg/m^2^ of straw, and after the mushrooms were harvested in April of the following year, all remaining cultivation substrates were rototilled *in situ* into the soil. The cultivation and management of *S. rugosoannulata* were performed according to the methods of Hao et al. (2024). After all the experimental treatments were completed, the rice was cultivated in June of the following year. Moreover, irrigation, fertilization and management methods during rice cultivation were carried out in the same way for the different treatments. The entire experiment was rotated continuously for 3 years, with sampling from 2020 to 2022. The 5-point method was used to collect soil samples from the rhizosphere of rice under CK, GM, RS and SRS treatments during rice elongation stage. Each treatment had 5 replicates, and all soil samples were divided into two groups, one for the determination of microbial diversity and one for the determination of soil physicochemical indices. The collected samples were stored at −80°C for subsequent experiments.

### Soil physicochemical properties and rice yield determination

In the rice planting area, five points (a total measurement area of 5 square meters) were randomly selected for rice threshing, drying and weighing to determine the yield. The soil organic matter (SOM), soil organic carbon (SOC), total nitrogen (TN), total phosphorus (TP), total potassium (TK), alkaline hydrolyzable nitrogen (AN), available phosphorus (AAP), available potassium (AK) and water-soluble total salt (WTS) levels were determined as described by Yue et al. (2024). In addition, Soil urease (S-UE) was determined at 578 nm using the indophenol-blue colorimetry method; Soil laccase was determined at 420 nm using the 2,2′-azinobis-(3-ethylbenzthiazoline-6-sulphonate) method; soil cellulase (S-CL) was determined at 620 nm using the anthrone colorimetric method; soil lignin peroxidase (S-Lip) was determined at 310 nm using the veratryl alcohol colorimetric method. Both soil saccharase (S-SC) and soil amylase (S-AL) were determined at 510 nm and 540 nm using the 3,5-dinitrosalicylic acid method, respectively. Each treatment assay was performed in 5 replicates.

### DNA extraction, sequencing process, and sequence analysis

According to the manufacturer’s protocols, microbial DNA was extracted from the CK, GM, RS and SRS soil samples by using a E.Z.N.A.^®^ Soil DNA Kit (Omega Biotek, Norcross, GA, United States). The V3–V4 regions of bacterial 16S rRNA and the internal transcribed spacer (ITS) of fungal rDNA genes from each extracted DNA sample were amplified using the primers 341F (CCTAYGGGRBGCASCAG)/806R (GGACTACN NGGGTATCTAAT) and ITS1F (CTTGGTCATTTAGAGGAAGTAA)/ITS4 (GCTGCGTTCTTCATCGATGC), respectively. Purified amplicons were pooled in equimolar, and paired-end sequenced using an Illumina MiSeq platform (Shanghai Biozeron Co., Ltd.). The raw reads were deposited into the NCBI Sequence Read Archive (SRP457894) database. After sequencing was completed, the reads were qualified, assembled and clustered into amplicon sequence variants (ASVs) using the DADA2 plug-in unit in QIIME2 software (Bokulich et al., 2018). All ASVs were assigned to a taxonomy based on the SILVA release 138 database (Yilmaz et al., 2014). Singletons (with one specific ASV) were removed, and the remaining data were normalized using the lowest read number among all samples.

### Statistical analysis

Soil basic properties and rice yields were analyzed using SPSS 20.0 and then plotted with GraphPad Prism 8. The statistical methods used were one-way analysis of variance and Tukey’s test. All microbial data analyses were performed by R v4.2.2 and visualized by the “ggplot2” package. The alpha diversity indices of the bacterial and fungal communities, including the Chao1 index, Shannon index, Pielou_J index and Pd_Faith index, were calculated (“Vegan” package). The niche breadth of the bacterial and fungal communities was measured based on Levins’ niche breadth index (R package “spaa”). A given taxon was categorized as a specialist or generalist based on the criterion *B* > 3 or *B* < 1.5, respectively (Xiong et al., 2020). Differences in the habitat niche breadth between bacterial and fungal communities were assessed by Student’s t test. The null model was used to identify the process governing microbial community assembly based on the βNTI and Raup–Crick metric (RC) (Stegen et al., 2013). Redundancy analysis (RDA) was performed with CANOCO5 software. Boxplots were drawn using the ggplot2 package in R software (Liu et al., 2021).

Furthermore, to decipher the distribution patterns of the soil bacterial communities, co-occurrence network analysis was performed using Cytoscape version 3.3.0 software with the CoNet plug-in (Faust and Raes, 2016). Pairwise correlations were determined using Pearson and Mantel correlations, mutual information, and Bray–Curtis and Kullback–Leibler dissimilarity tests. The network graph was visualized using the Gephi interactive platform, displaying only correlations with a *p*-value >0.8 and a *p*-value <0.05. The topological roles of individual nodes in the networks were evaluated by the threshold values of Pi and Zi (Olesen et al., 2007). The relationships between the keystone of the co-occurrence network and environmental variables were measured by Spearman correlation. Structural equation modeling (SEM) was performed with AMOS 21.0 using the maximum likelihood estimation method. The fit of the suitable model was judged by the nonsignificant chi-square test (*p* > 0.05), high goodness-of-fit index (GFI >0.90), and low root mean square errors of approximation (RMSEA <0.05) (Hao et al., 2024). All figures were combined and processed with Adobe Illustrator 2023.

## Results

### Response of soil physicochemical properties and rice growth to SRS return

**Figure 1:**
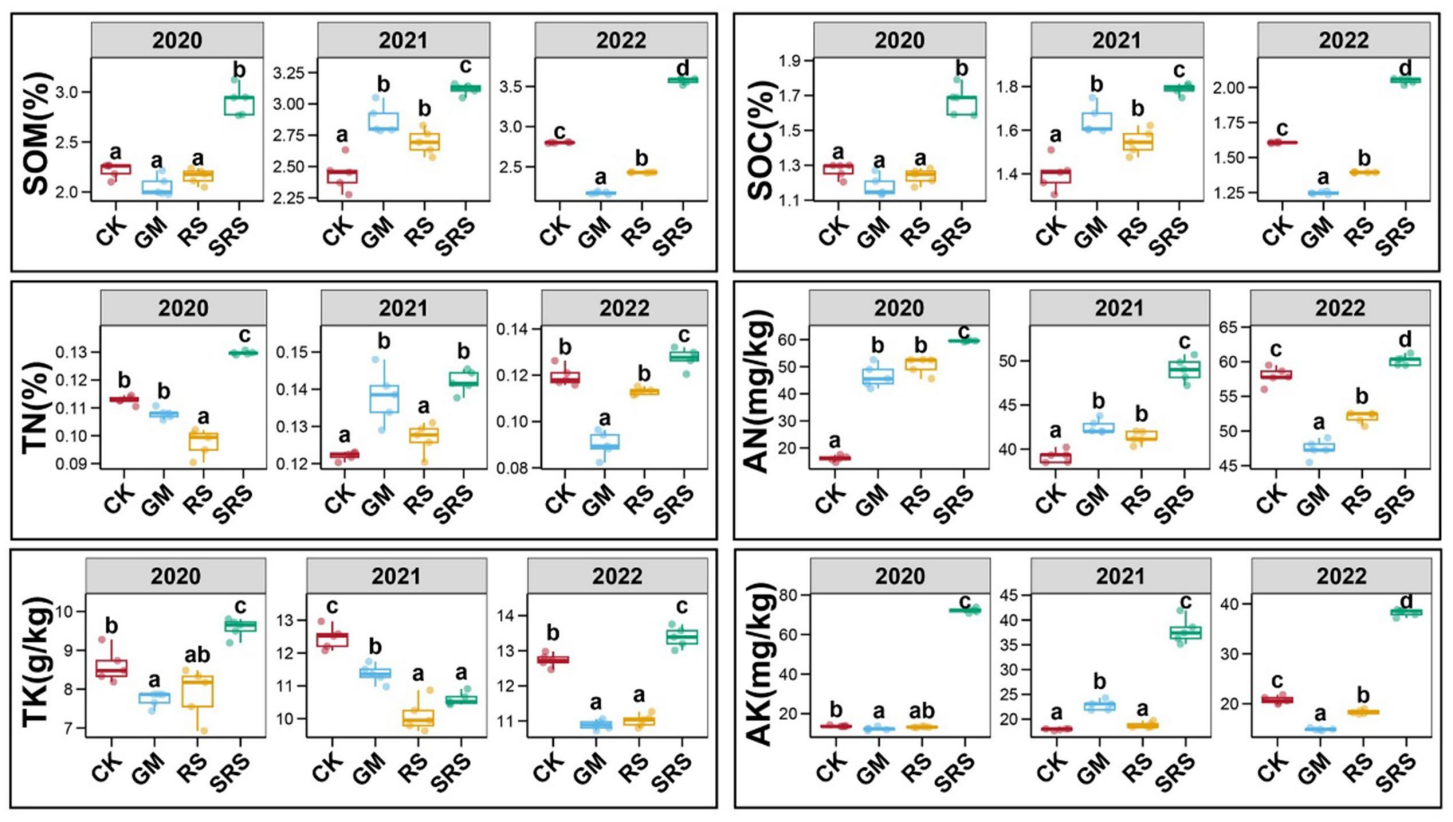
Comparison of soil physicochemical indices, enzyme activities and yield between different treatments. The soil physicochemical indices included soil organic matter (SOM), soil organic carbon (SOC), total nitrogen (TN), alkaline hydrolyzable nitrogen (AN), total potassium (TK) and available potassium (AK). Error bars represent the standard deviation of the mean. Numbers in a rank with different letters indicate a significant difference (Tukey’s test, *p* < 0.05).

Crops need soil to provide nutrients for growth, of which soil organic matter (SOM), soil organic carbon (SOC), nitrogen (N), phosphorus (P) and potassium (K) play vital roles in increasing rice yields. Figure 1 shows that SRS return significantly improved the nutrient content of the paddy soil. From 2020 to 2022, the return of SRS to the field increased SOM and SOC by 27.0–31.6% and 27.4–30.6%, respectively. Most importantly, when SRS was returned to the field, the available nitrogen (AN) and available potassium (AK) increased by 5.3–272.3% and 83.8–421.7%, respectively (Figure 1). Moreover, total nitrogen (TN) and total potassium (TK) increased in most years. However, we found that soil total phosphorus (STP) and available phosphorus (SAP) did not change significantly after SRS treatment (Supplementary Figure S2). In addition, we investigated the traditional RS return and GM return patterns, and the results showed that the contents of SOM and SOC under the two patterns were greater than those in the control group in 2021. The AN content of the GM and RS treatments was greater than that of the control group in 2020 and 2021 but lower than that of the SRS treatment group, and the RS treatment increased the content of SAP. The water-soluble total salt content and pH under the various treatments differed, and the water-soluble salt content under the various treatments was greater than that under the control treatment in 2022, while the pH was lower than that under the control treatment (Supplementary Figure S2).

Soil enzyme activity is also an important indicator of soil health. We found that soil urease and soil lignin peroxidase activities increased significantly by 36.2–98.3% and 27.5–64.8%, respectively, after the SRS treatment in 2020 and 2021 (Supplementary Figures S3A,E). Compared with those in the control treatment, the soil sucrase, amylase and laccase activities increased by 19.1–116.5%, 15.4–35.3% and 103.9–771.5%, respectively, in the three different years of the mushroom-rice rotation (Supplementary Figures S3B,D,F). There was no significant change in soil cellulase activity in the various treatments (Supplementary Figure S3C). We found that, compared with the control treatment, the SRS treatment increased the rice yield by 13.0% in the first year. In the second and third years of the mushroom–rice rotation, compared with the control treatment, the SRS treatment significantly increased the rice yield by 15.9 and 19.9%, respectively (Supplementary Figure S3G). The return of green manure to the field increased the rice yield by 3.7 and 4.5% in the second and third years, respectively (Supplementary Figure S3G). However, straw return reduced rice yields, but there was no significant difference in yield compared with that of the control in the third year. In general, the SRS treatment accelerated the accumulation of soil nutrients by enhancing soil enzyme activity, thereby increasing rice yield.

### Effects of SRS return on soil community diversity and composition

**Figure 2:**
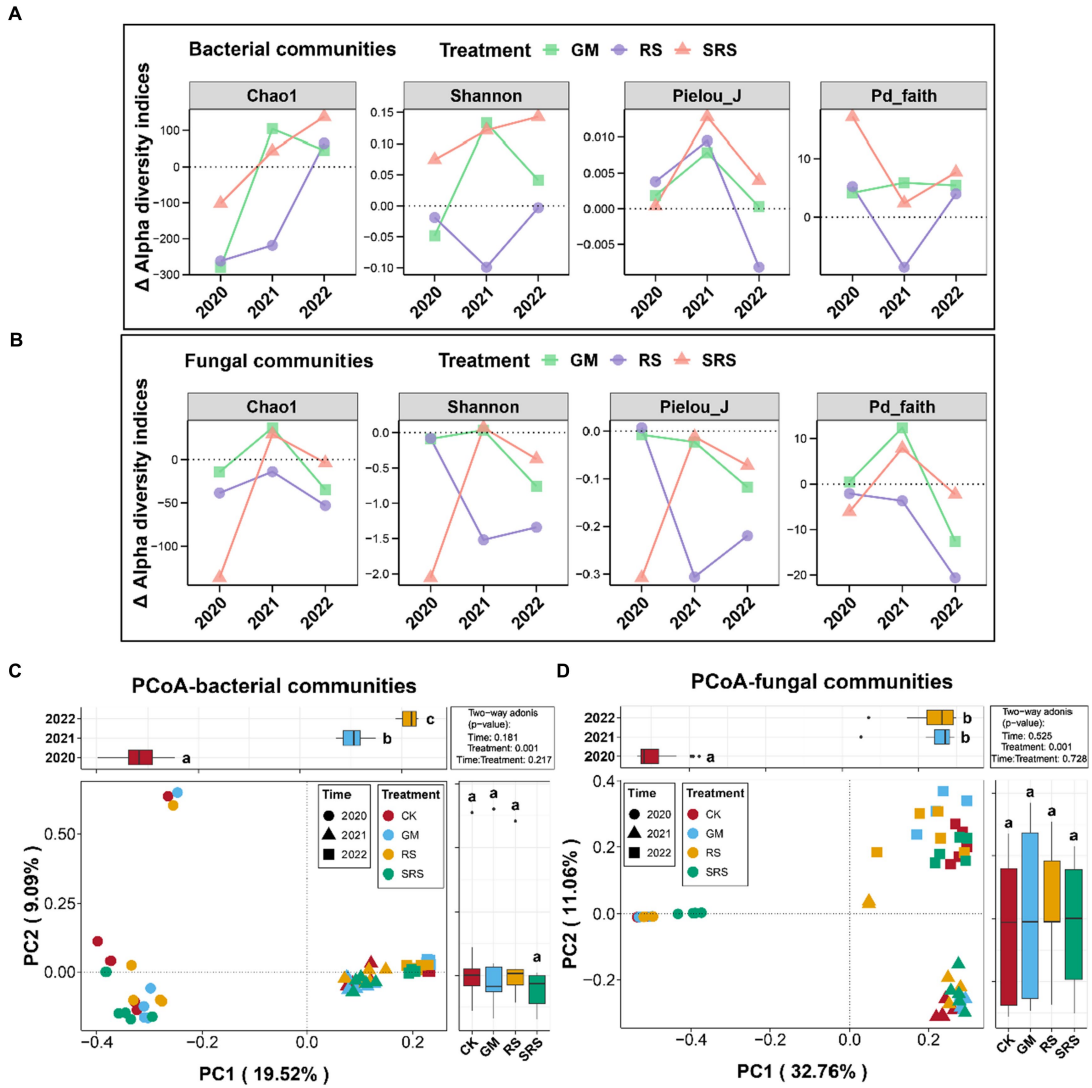
Community variations in bacterial alpha diversity **(A)** and fungal alpha diversity **(B)** of the paddy soil samples at the interannual level. Principal coordinate analysis (PCoA) and PERMANOVA of the bacterial **(C)** and fungal **(D)** communities in the paddy soils. Above and right boxplots represent the values of samples from different years and treatments on PC1 and PC2, respectively. Different lowercase letters above each box in the same subfigure represent significant differences between groups (Tukey’s test, *p* < 0.05).

The alpha diversity indices of the 4 treatments (CK, GM, RS, and SRS) were calculated for 3 consecutive years. No significant differences were found in the Shannon, Simpson, Pielou_J or Pd_faith indices of the bacterial diversity among the different treatments in most years (Supplementary Figure S4). The fungal alpha diversity index of the SRS treatment was lower than that of the other treatments in the first year, and there was no significant change in the other years (Supplementary Figure S5). We further analyzed the interannual relative alpha diversity trends of the GM, RS and SRS treatments and found that the relative alpha diversity index of bacteria in the SRS treatment was greater than that in the other treatments in most years (Figure 2A). However, the relative alpha diversity of fungi in the different treatment groups was lower than that in the control group (Figure 2B).

PCoAs based on the Bray–Curtis distance were used to assess the spatial variation in bacterial and fungal compositions in the paddy soil, and the results are shown in Figure 2. The first two PCs accounted for 28.61% of the total variation in the bacterial communities. For fungal communities, the first two PCs explained 43.76% of the total variation. Moreover, the PERMANOVA tests demonstrated that the interannual level had a significant impact on the bacterial and fungal communities (*p* < 0.05), but there was no significant difference between treatments. We found that the bacteria and fungi in the different interannual samples were distributed along the PC1 axis, and the bacteria and fungi in the samples from the same year were relatively similar (Figures 2C,D).

### Effects of SRS return on soil microbial community abundance

**Figure 3:**
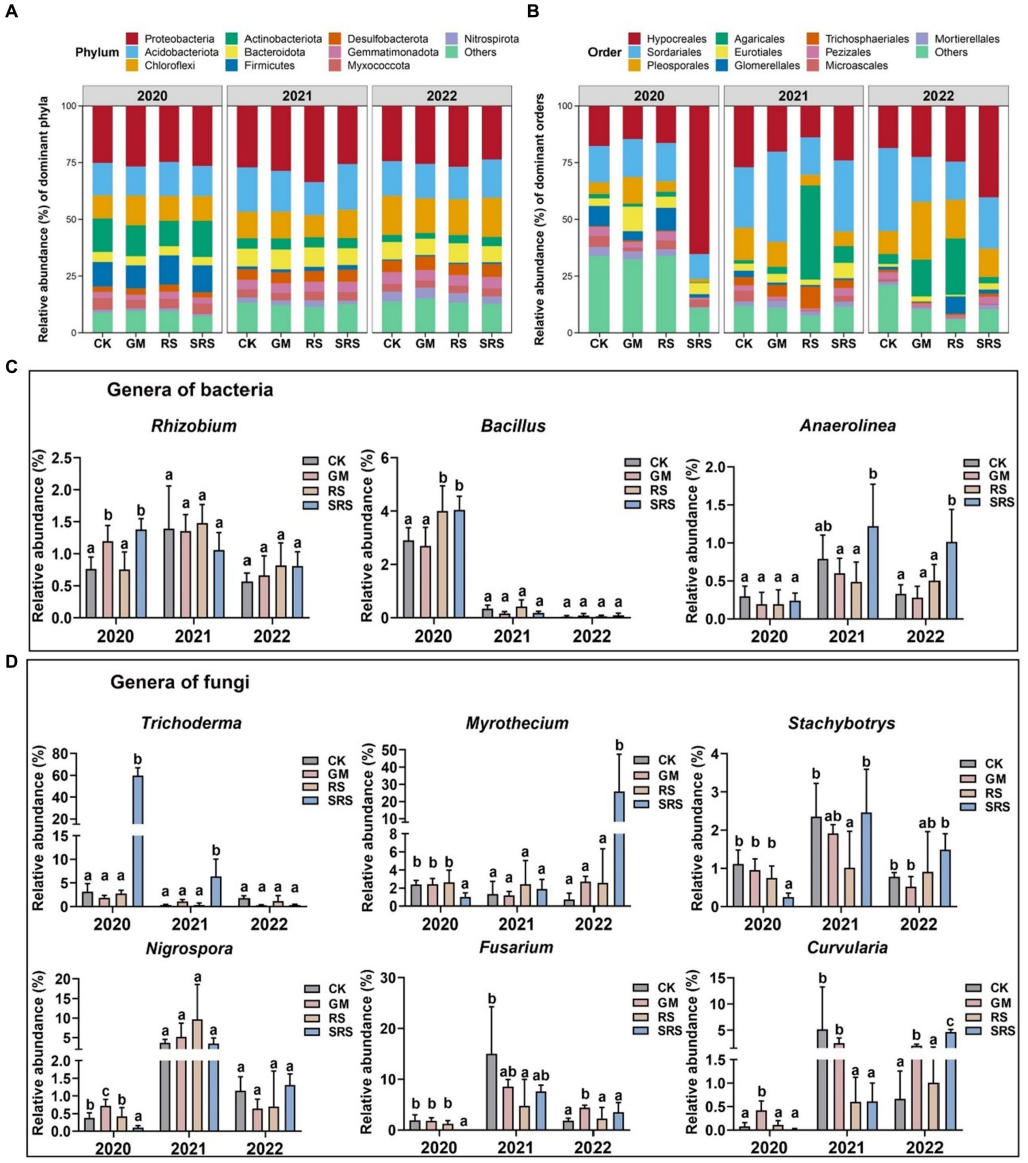
Relative abundance at the level of the bacterial **(A)** phylum and at the level of the fungal **(B)** order of soils from different treatments, as well as the relative abundance of microorganisms with large bacterial **(C)** and fungal **(D)** differences at the genus level. Different lowercase letters represent significant differences (Tukey’s test, *p* < 0.05).

The bacterial communities of paddy soil samples were dominated by the phylum Proteobacteria, followed by Actinobacteria, Chloroflexi, Acidobacteriota, Firmicutes, and Myxococcota (Figure 3A). There was little variation at the bacterial phylum level among the different treatments. The majority of fungal ASVs belonged to Ascomycota and Basidiomycota. The abundance of Ascomycota (88.19–95.7%) after the SRS treatment was greater than that after the other treatments. Significant changes in fungal communities at the order level were also detected after SRS treatment, and Hypocreales, Sordariales, Pleosporales, Agaricales, and Eurotiales were also dominant (Figure 3B).

We found that the abundance of SRS-treated *Bryobacter* increased by 33.6 and 3.6% relative to that of the controls in 2020 and 2021, respectively (Supplementary Figure S6). However, the abundance of *Nitrospira* was significantly lower, by 29.6–48.4%, for 3 consecutive years after SRS treatment (Supplementary Figure S6). The abundances of the fungal genera *Acremonium*, *Chytriomyces*, *Mortierella* and *Humicola* were lower in the treated group than in the control group in different years (Supplementary Figure S7). In addition, we focused on *Rhizobium* among the soil nitrogen-fixing microorganisms and found that its abundance significantly increased by 50.9 and 80.8% in the first year of the GM and SRS treatments, respectively (Figure 3C). It was also found that the abundances of RS- and SRS-treated *Bacillus* plants improved by 27.6 and 28.3%, respectively, in the first year (Figure 3C). The abundance of *Anaerolinea* significantly increased after SRS treatment in 2021 and 2022 (Figure 3C). Among the fungi, *Trichoderma* was significantly enriched after SRS treatment, which was 18-fold greater than that in the other treatments in 2020 and 2021 (Figure 3D). SRS treatment resulted in a decrease in the abundance of both *Myrothecium* and *Stachybotrys* in 2021 but also a significant increase in 2022 (Figure 3D). Among the pathogenic fungi, we also focused on changes in the abundances of *Nigrospora*, *Fusarium*, and *Curvularia* and found that, compared with the other treatments, the SRS treatments decreased their abundance in 2020 (Figure 3D). The abundances of *Fusarium* and *Curvularia* were also lower than those in the control treatment in 2021, but showed the opposite trend in 2022.

### Effects of different returning treatments on soil microbial assembly in paddy fields

The relationships between the detection rates of bacteria and fungi in the soil samples with different returning patterns and their average relative abundances were estimated (Supplementary Figure S8). There was a significant correlation for both bacteria and fungi, and the correlation coefficient (*r*^2^ = 0.581) for fungi was greater than that for bacteria (Supplementary Figures S8A,B). Furthermore, bacteria and fungi were classified into persistent species, intermediate species and transient species, and the differences in their numbers and abundances were analyzed. Most bacterial and fungal species were transient species, accounting for 94.06 and 92.36%, respectively, while the proportions of persistent species were only 0.37 and 0.00%, respectively (Supplementary Figures S8C,D).

**Figure 4:**
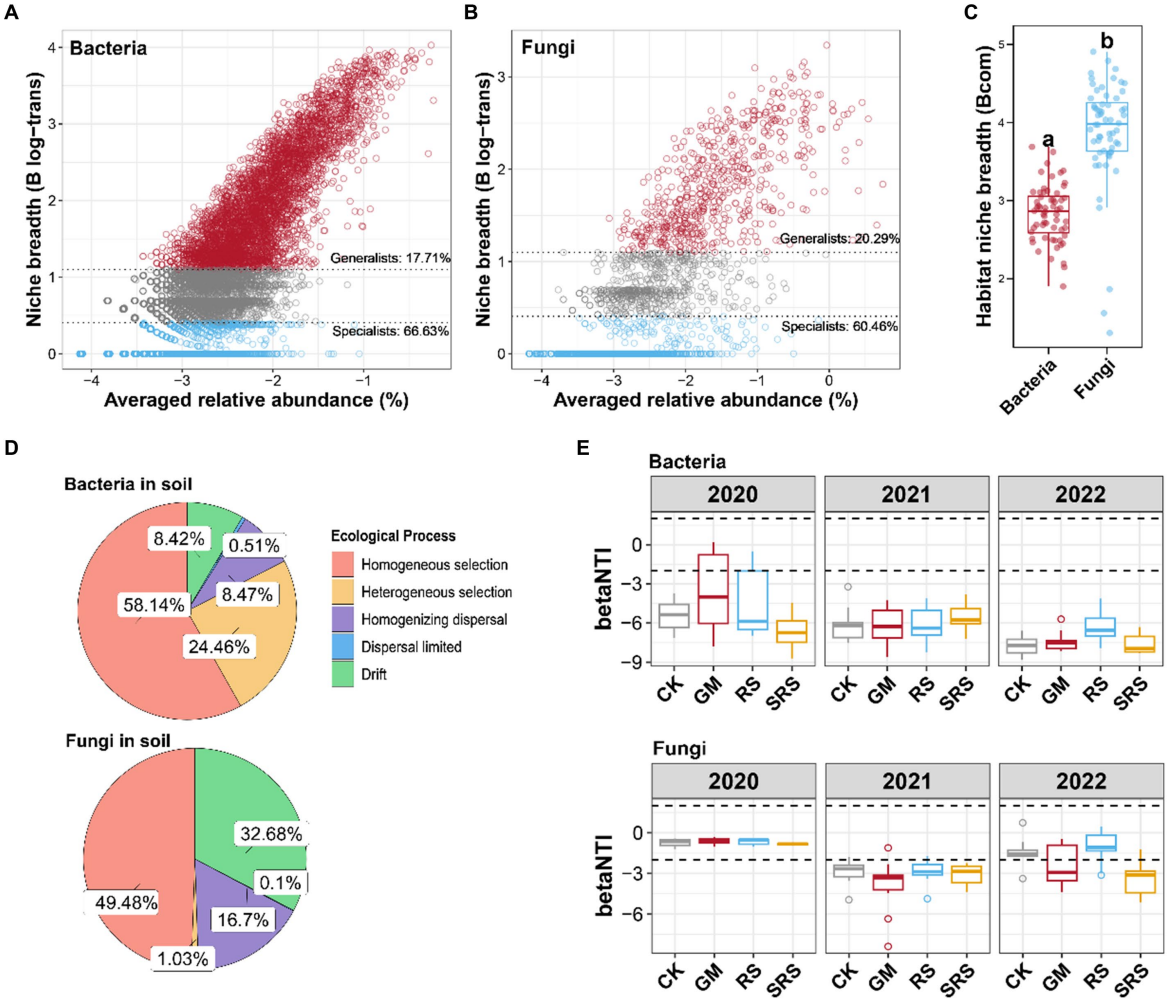
The assembly mechanism of the bacterial and fungal communities in the paddy soil samples. **(A)** Relationship between niche breadth and the relative abundance of bacteria. **(B)** Relationship between niche breadth and the relative abundance of fungi. **(C)** Boxplots illustrating the mean habitat niche breadth from all taxa in each sample of bacterial versus fungal communities. **(D)** Proportion of bacterial and fungal assembly types. **(E)** The β-NTI values of the bacteria and fungus. Different lowercase letters represent significant differences (Tukey’s test, *p* < 0.05).

The bacterial and fungal species were further divided into generalists and specialists (Figures 4A,B). Compared with the fungal community, the bacterial community had a lower proportion of generalists and a greater proportion of specialists. Moreover, the niche width analysis revealed that the bacterial community had a lower niche width than did the fungal community (Figure 4C). The ecological process analysis showed that bacteria were mainly shaped by the homogeneous selection process, while the contribution of heterogeneous selection in the shaping process of fungi was very small, and most fungi were contributed by ecological drift and homogeneous diffusion (Figure 4D). The null model was used to explore the assembly mechanism of the bacterial and fungal communities. Most β-NTI values for the bacterial community were less than −2 in the soils subjected to the different treatments, and there were no significant differences between the SRS-treated and GM- and RS-treated groups. The β-NTI of fungal communities decreased first and then increased in the control, GM and RS treatment groups, while that in the SRS treatment group decreased and was lower than −2 (Figure 4E). These results indicated that the three-year SRS rotation changed the soil fungal community from a stochastic process to a deterministic selection process.

### Relationships between soil physicochemical properties and the microbial community

**Figure 5:**
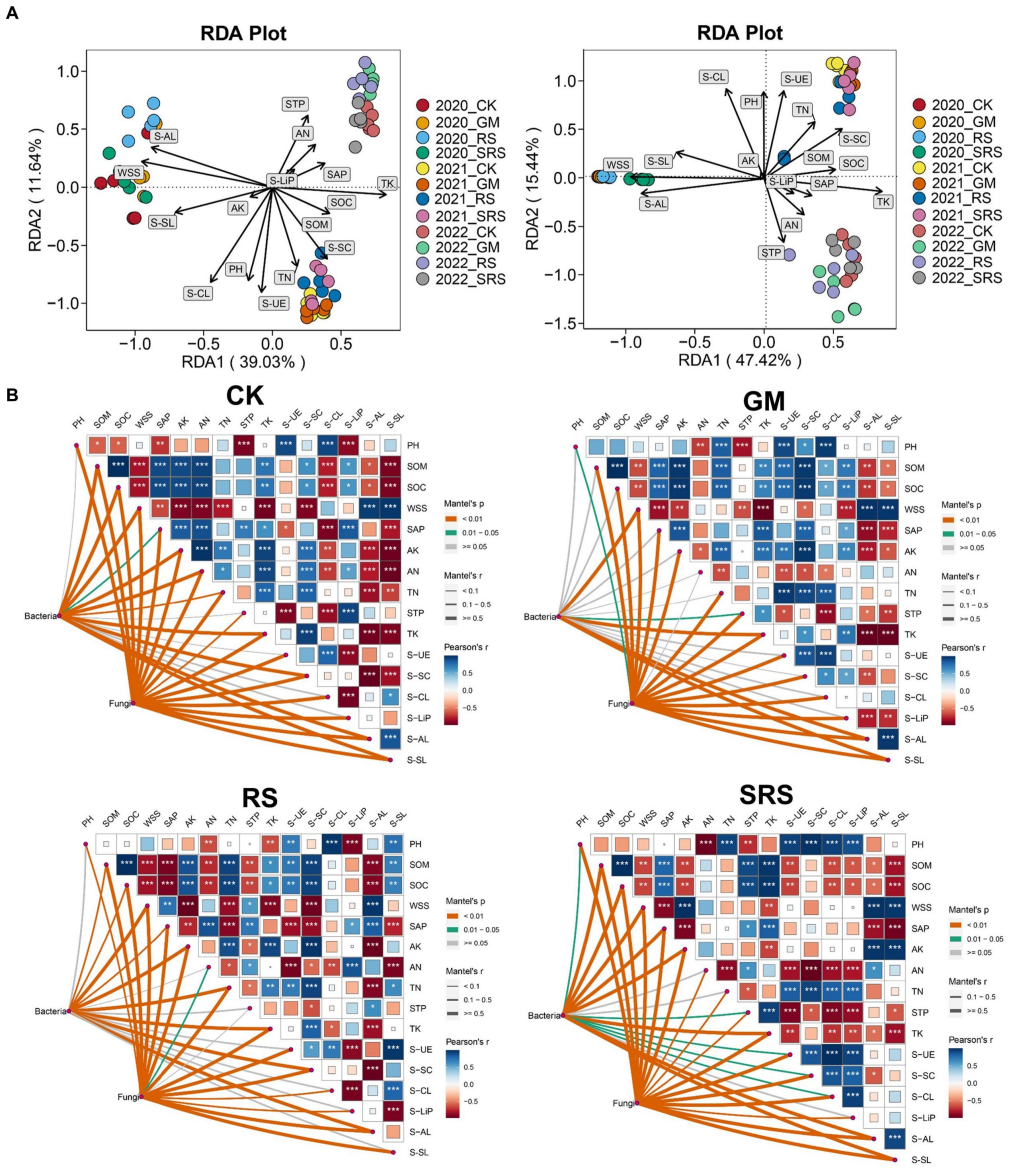
RDAs between the bacterial community and fungal community with respect to soil physicochemical factors. **(A)** Correlation analyses between the bacterial communities and soil physicochemical properties during the SRS return to the field using the Mantel test. **(B)** SOM, soil organic matter; SOC, soil organic carbon; TN, total nitrogen; AN, alkaline hydrolyzable nitrogen; TK, total potassium; AK, available potassium; STP, soil total phosphorus; SAP, soil available phosphorus; WSS, water-soluble salt; S-UE, soil urease; S-SC, soil saccharase; S-CL, soil cellulase; S-AL, soil amylase; S-Lip, soil lignin peroxidase; S-SL, soil laccase. ^*^0.01 ≤ *p* < 0.05, ^**^0.001 ≤ *p* < 0.01, ^***^p < 0.001.

RDA was used to evaluate the relationships between physicochemical indicators (soil nutrients and enzyme activity) and microbial communities in the various treated soils. The RDA1 and RDA2 axes explained 39.03 and 11.64% of the changes in the bacterial community and 47.42 and 15.44% of the fungal community changes, respectively (Figure 5A). Moreover, the nutrients WSS, TK, TN, STP, SOM and SOC, as well as the S-AL, S-CL and S-UE enzymes, had a great effect on the fungal and bacterial communities. Furthermore, Mantel tests were used to compare the correlation between physicochemical properties and the relationship with microbial flora between different treatments, and the results showed that most of the soil physicochemical indices and enzyme activities were significantly correlated with different treatments. It is worth noting that AN and TN in the SRS treatment group were not correlated with SOM or SOC (Figure 5B). In addition, most of the physicochemical indices in the SRS treatment group were closely related to bacteria, followed by those in the control group, and the GM treatment group had the lowest correlation. At the same time, the relationships between fungi and physicochemical indices were very close among the treatment groups, and there was no significant difference (Figure 5B).

**Figure 6:**
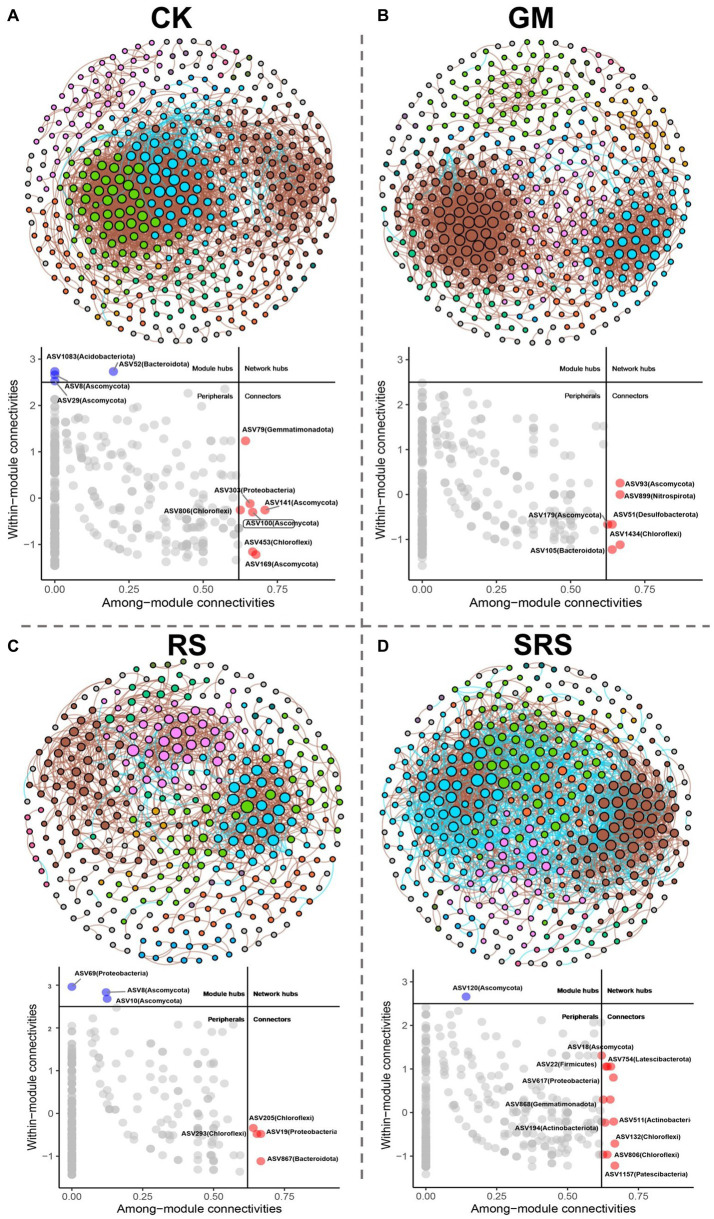
Fungal-bacterial co-occurrence network and Zi-Pi chart analysis of soil after control **(A)**, green manure return **(B)**, straw return **(C)** and *S. rugosoannulata* cultivation substrate return **(D)**.

To compare the correlations between microorganisms, a fungal-bacterial co-occurrence network was constructed for the different treatment groups, and the network between the different treatment groups exhibited good modularity characteristics. Most of the modules exhibited a variety of different species. The whole network of the control group included 324 nodes and 1731 edges (Figure 6). Compared to the control group, the RS-treated group had the least number of nodes and edges at 268 and 837, respectively, while the SRS-treated group had the most nodes and edges at 395 and 2,604, respectively (Figure 6). The key species in the network were identified by Zi-Pi analysis, and 4 module hubs and 7 connectors were identified in the control group, which may have played an important role in the whole bacterial-fungal network (Figure 6D). Six connectors were identified in the GM treatment group, 3 module hubs and 4 connectors were identified in the RS treatment group, and 1 module hub and 13 connectors were identified in the SRS treatment group. More key bacteria were associated with the network after SRS treatment (Figure 6). Spearman correlation analysis was used to evaluate the relationships between the abundances of these key species and nutrient composition and soil enzyme activity (Supplementary Figure S9). Compared to other treatment groups, the SRS treatment group had more keystone species, Latescibacterota (ASV120, ASV754), Chloroflexi (ASV806), Gemmatimonadota (ASV868), Patescibacteria (ASV1157), and Ascomycota (ASV18, ASV120), were positively correlated with physicochemical indices.

**Figure 7:**
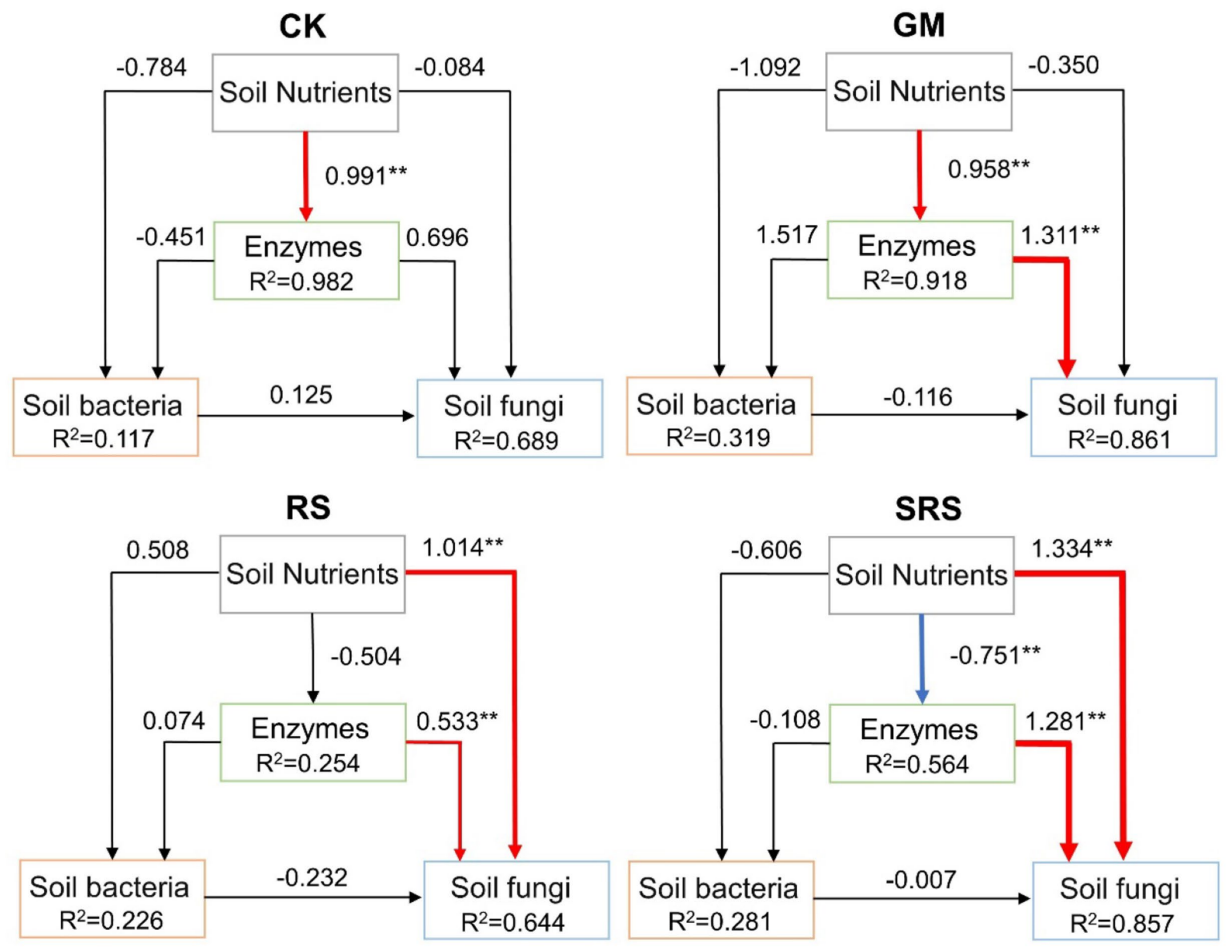
Structural equation models reflecting the soil nutrients, soil enzymes and microbial diversity. The numbers adjacent to the arrows are path coefficients. The red line represents the positive path coefficient, the blue line represents the negative path coefficient, and the black line represents the nonsignificant path coefficient. The width of the arrow indicates the significance of the standard path coefficient (^**^p < 0.05).

SEM was performed to evaluate the effects of soil nutrients (SOC, SOM, TN, AN, TK, AK, STP, SAP, WSS, pH) and enzyme activities (S-UE, S-SC, S-CL, S-Lip, S-AL, S-SL) on fungal and bacterial communities (Figure 7). The results showed that soil nutrients in the control group had a positive effect on enzyme activity, while soil enzymes in the GM treatment group had a positive effect on fungal communities (Figure 7). The soil nutrients and enzyme activities in the RS and SRS treatments had a positive effect on the fungal community (Figure 7). In addition, soil nutrients after SRS treatment had a negative effect on soil enzymes.

## Discussion

### Impact of SRS return on soil physicochemical properties and rice yield

In traditional agricultural production, straw treatment methods and soil improvement solutions have several limitations, and emerging solutions need to be explored (Ma et al., 2020). In this study, winter fallowing (CK), RS return, and GM return were used as references to fully explore the effects of SRS return on rice field soils. Compared with the other treatments, 3 years of rice-mushroom rotation significantly increased the contents of soil SOC, SOM, N and K (Figure 1). Previous rice-wheat and rice-green manure rotations also increased the contents of SOC, AN and AK, which is consistent with our results (Feng et al., 2020). We found that the SRS had little effect on phosphorus. The RS treatment increased the accumulation of phosphorus in the soil, while green manure return reduced phosphorus. This may be caused by differences in a plant’s ability to absorb phosphorus (Supplementary Figure S2). Excess soluble salts have toxic effects on plant growth (Liu et al., 2023), and although the soluble salts in the SRS treatment were greater than those in the other treatments, they were all within a reasonably safe range. This suggests that legume crops and SRS are rich in nutrients, so returning them to the field can significantly improve soil fertility, while the mycelia of *S. rugosoannulata* were the most effective at efficiently decomposing straw, and SRS improved the soil.

Soil enzyme activity can indicate the impact of soil management on processes such as soil decomposition and nutrient cycling (Balota et al., 2004). Compared with those of the control, the soil urease and lignin peroxidase enzyme activities increased by more than 27% in the first 2 years after SRS treatment, and the soil amylase and laccase activities also increased significantly, which was similar to the results of other crop rotation methods. Previous studies revealed that rice-green manure had little effect on sucrase or cellulase, which is consistent with our findings for the GM treatments (Figures 3A,C). We found that the rice yield of the rice-mushroom rotation system was the highest, followed by that of the rice-green manure system (Supplementary Figure S3G), indicating that the physical and chemical indices of the soil improved in response to crop rotation and improved the rice yield, and the rice-mushroom rotation pattern had the greatest effect.

### Impact of SRS return on soil diversity and the composition of the microbial community

Our results showed that different treatments did not have much effect on the bacterial alpha diversity index (Supplementary Figure S4) but reduced the fungal alpha diversity index in the short term (Supplementary Figure S5). The understory cultivation of *S. rugosoannulata* also had little effect on the Shannon and Simpson indices of soil alpha diversity (Gong et al., 2018). We further analyzed the magnitude of bacterial changes in different treatments relative to the control at the interannual level, and the results showed that the changes in the Chao1 and Shannon indices continued to increase after SRS treatment. The relative alpha diversity index of the GM treatment first increased and then decreased, while that of the RS treatment showed the opposite trend (Figure 2A). These results indicated that SRS return had the greatest positive effect on bacterial diversity at the interannual level. Previous studies have shown that the excessive input of spent mushroom substrate into the field reduces the alpha diversity index of the microbial community (Li et al., 2020). This provides information about the rice-mushroom rotation pattern in which SRSs are returned to the field to improve the diversity of soil microbial communities, but the accumulation of organic matter in SRSs may have a negative impact on microbial diversity.

PCoA was performed based on the Bray–Curtis distance, weighted UniFrac distance and unweighted UniFrac distance (Zhang et al., 2021). The total interpretation rate of PCoA was greater in both the bacterial and fungal communities, indicating that species richness among the different treatments was one of the main reasons for the structural differences in the microbial communities at the interannual level (Figures 2C,D). Studies have shown that adequate nutrition can increase microbial diversity (Liu et al., 2017). This indicates that the soil microbial structure of paddy fields is relatively stable and that the accumulation of exogenous organic matter inputs has an important impact on β diversity. We found that the abundances of Rhizobia and Bacillus in the SRS-returned soil increased in different years (Figure 3C). Rhizobia species are considered the most effective microorganisms for assisting plants in nitrogen fixation, while Bacillus species not only have the ability to fix nitrogen but also have metabolites that inhibit the growth of pathogenic microorganisms (Hoang et al., 2022). Therefore, the increase in the abundance of nitrogen-fixing microorganisms may be a factor in soil nitrogen accumulation. Among fungi, the abundance of *Trichoderma* increased dozens of times in the first 2 years of rice-mushroom rotation (Figure 3D), and it has also been used as a fungal biocontrol agent to inhibit the growth of pathogenic microorganisms (Manzar et al., 2022). In addition, we focused on soil phytopathogenic bacteria such as *Nigrospora* (Hao et al., 2020), *Fusarium* (Haidukowski et al., 2022), and *Curvularia* (Marin-Felix et al., 2020), which cause crop yield reductions. We found that the abundances of these pathogenic fungi decreased in response to the rice-mushroom rotation in the first 2 years but had little effect in the third year (Figure 3D). Therefore, suitable durations of rice-mushroom rotation are crucial for regulating the abundance of plant pathogenic fungi.

### Effects of SRS returned to the field on the ecological process of soil microbial community assembly

Studies have shown that the deterministic assembly process of bacteria in wetlands and forests plays an important role in the processes of ecosystems (Mendes et al., 2015; Wang et al., 2021). Our study also revealed that soil bacteria after rice-mushroom rotation were affected by deterministic processes, similar to the conclusions of previous studies. However, fungi were affected by stochastic processes (Figure 4D). In China’s forest ecosystem, the assembly of bacterial and fungal communities is dominated by stochastic and deterministic processes, respectively, which is contrary to our conclusion, and in forest ecosystems, warming can shift bacterial assembly from stochastic to deterministic processes (Zhou et al., 2023); therefore, environmental factors affect the assembly process of microorganisms. This suggests that the transfer of organic matter from rice-mushroom rotations to the soil plays a driving role in microbial assembly.

### SRS-mediated soil nutrient changes and microbial succession regulate soil health

The relationships between soil physicochemical properties and microorganisms are mutually influential, with soil microorganisms altering the environment by changing the properties of surrounding soils or constructing mineral structures, and soil physicochemical properties also affect soil microbial communities and compositions (Philippot et al., 2023). In our study, the rice-mushroom rotation had a significant positive effect on the soil physicochemical properties. Mantel tests revealed that most of the soil physicochemical indices after rice-mushroom rotation were significantly correlated with the abundances of bacteria and fungi (Figure 5). The short-term application of spent mushroom substrate to soil showed that microorganisms were closely related to soil physicochemical indices (Chen et al., 2022), which is consistent with our results. In addition, we found a significant positive correlation between soil physicochemical properties (SOM, SOC, TN and AK) and enzyme activities (S-UE, S-SC and S-LiP) (Figure 5B). An analysis of different land use patterns revealed that five soil enzymes were involved in the carbon, nitrogen and phosphorus cycles and their responses to changes (Xie et al., 2017). These results indicated that soil enzyme activity could be used as one of the key indicators for evaluating and predicting soil quality and that rice-mushroom rotation had a good effect on maintaining soil nutrients and health.

Co-occurrence network analysis revealed that bacteria had more connection points and modules than fungi during the composting process of the spent mushroom substrate, which means that the spent mushroom substrate had a greater impact on bacteria (Qian et al., 2022), which is consistent with our conclusion. We found that Latescibacterota, Chloroflexi, Gemmatimonadota and Patescibacteria had the greatest positive effects on soil properties and enzyme activity among the microorganisms (Supplementary Figure S9D). Latescibacterota has been shown to degrade complex plant polysaccharides (Wang et al., 2024). Chloroflexi are widely distributed in various biosphere environments and participate in the ecological cycles of C, N and S in soil and compost habitats (Sorokin et al., 2014), while Gemmatimonadota is very rich in soil and can degrade organic substrates (Zheng et al., 2022), and Patescibacteria play an important role in the ecosystem (Tian et al., 2020). These results suggest that the rice-mushroom rotation pattern can jointly promote the sustainable and healthy development of paddy ecosystems through interactions between a few key microorganisms and soil physical and chemical properties.

The effects of soil nutrients and enzyme activities on bacterial and fungal diversity were evaluated by SEM. Previous studies have shown that higher nutrients in the soil have a negative effect on fungal diversity (He et al., 2016), while we found that rice-mushroom rotation has a positive effect on soil fungi, which may be related to the uptake and utilization of nutrients by rice during crop rotation. Nutrients had a greater effect on bacterial diversity during the return of spent mushroom substrate to the forest (Hao et al., 2024), which is inconsistent with our results and may be related to the cyclical changes in nutrients in the crop rotation system. Notably, soil physicochemical properties are closely related to the key fungal species ASV8 and ASV120 (Supplementary Figure S9D), which may also indicate that soil nutrients affect fungal community construction through key species. Therefore, the interaction between soil properties and microorganisms after rice-mushroom rotation promoted soil nutrient cycling and promoted interactions between microorganisms, which helped to maintain soil microbial diversity in paddy fields.

## Conclusion

In this study, the effects of SRS return on rice yield, soil physicochemical properties and the microbial community were fully explored and compared with those of traditional fallow, straw return and green manure return. Our experimental results demonstrated that rice yield, soil nutrients and soil enzyme activities positively responded to the rice-mushroom rotation pattern compared to the traditional crop rotation pattern. In terms of microorganisms, the rice-mushroom rotation model increased the relative alpha diversity index of bacteria at the interannual level and enriched nitrogen-fixing and biocontrol microorganisms such as *Rhizobium*, *Bacillus* and *Trichoderma*. Moreover, analysis of the microbial assembly mechanism in the rice-mushroom rotation system revealed that bacteria were affected by deterministic processes, while fungi were affected by stochastic processes. There was a significant correlation between soil nutrients and enzyme activities, as well as between soil physicochemical properties and microbial richness during the rice-mushroom rotation. Eleven key microorganisms closely related to the soil physicochemical properties were identified under the rice-mushroom rotation system. SEM showed that soil nutrients and enzyme activities had significant effects on fungal community construction. These results contribute to our understanding of the relationships between soil properties and microbial communities in paddy fields in rice-mushroom rotation systems. This study provides new insights into the involvement of edible mushrooms in the sustainable development of ecological agriculture.

## Data availability statement

The datasets presented in this study can be found in online repositories. The names of the repository/repositories and accession number(s) can be found in the article/Supplementary material.

## Author contributions

HH: Data curation, Formal analysis, Investigation, Methodology, Project administration, Software, Validation, Writing – original draft. YY: Methodology, Software, Writing – review & editing. QW: Methodology, Software, Writing – review & editing. TX: Methodology, Software, Writing – review & editing. ZZ: Methodology, Software, Writing – review & editing. JZ: Methodology, Software, Writing – review & editing, Funding acquisition, Project administration. HC: Funding acquisition, Project administration, Supervision, Writing – review & editing.
