# Supplementary material for: Effects of the rice-mushroom rotation pattern on soil properties and microbial community succession in paddy fields
Source: Front Microbiol. 2024 Jul 24;15:1449922. doi: 10.3389/fmicb.2024.1449922 (PMC11303333; doi:10.3389/fmicb.2024.1449922)
Supplement: Supplementary file 1 [file Data_Sheet_1.pdf]

Supplementary Information for

Effects of the Rice-Mushroom Rotation Pattern on Soil Properties and Microbial Community Succession in Paddy Fields

Haibo Hao [a, b], Yihong Yue [a], Qian Wang [a], Tingting Xiao [a], Zelong Zhao [c], Jinjing Zhang [a*] and Hui Chen [a*]

[a] National Research Center for Edible Fungi Biotechnology and Engineering, Key Laboratory of Applied Mycological Resources and Utilization, Ministry of Agriculture, Shanghai Key Laboratory of Agricultural Genetics and Breeding, Institute of Edible Fungi, Shanghai Academy of Agricultural Sciences, Shanghai 201403, China

[b] State Key Laboratory of Genetic Engineering and Fudan Center for Genetic Diversity and Designing Agriculture, Institute of Plant Biology, School of Life Sciences, Fudan University, Shanghai 200438, China

[c] Shanghai BIOZERON Biotechnology Co., Ltd., Shanghai, 201800, China

*Corresponding author: E-mail addresses:

Jinjing Zhang: 20150569@saas.sh.cn

Hui Chen: chenhui@saas.sh.cn

Full postal address: 309 Room, Institute of Edible Fungi, No. 1000, Jinqi Road, FengXian District, Shanghai 201403, China

Supplementary Figures

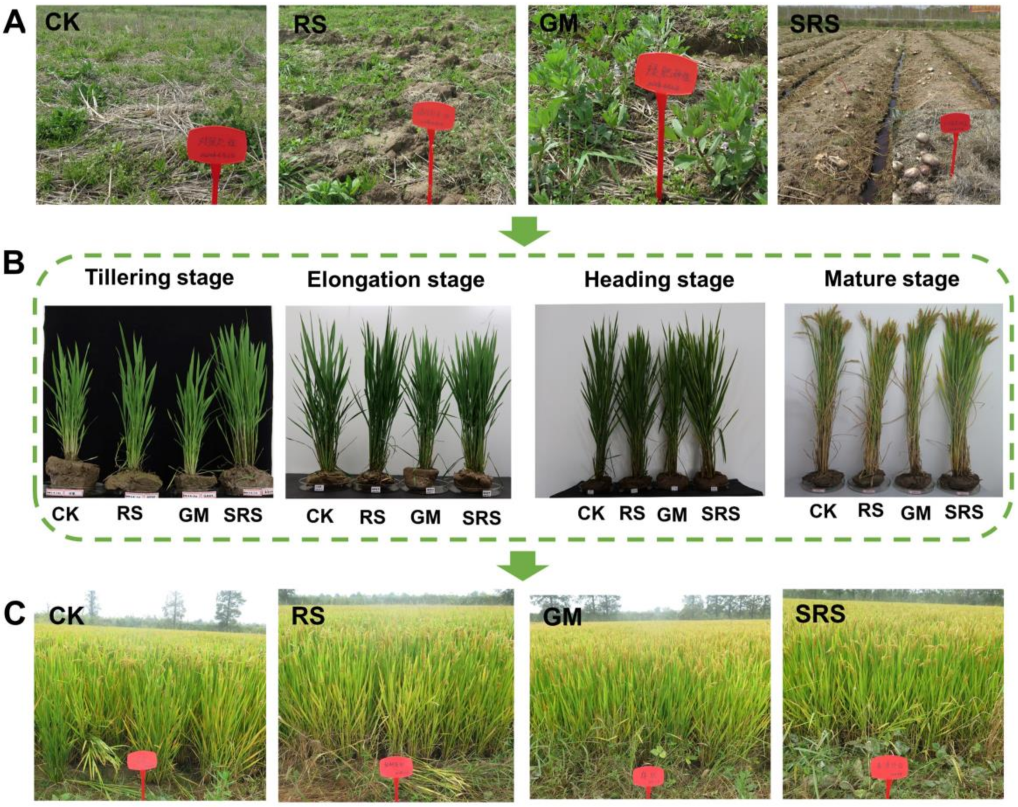

FIGURE S1 The field of different return treatments after rice harvest in autumn (A), the observation of the different growth and development stages of planted rice (B), and the harvest of rice after different treatments (C). CK: no treatment, RS: rice straw return, GM: green manure return, SRS: *Stropharia rugosoannulata* cultivation substrate return.

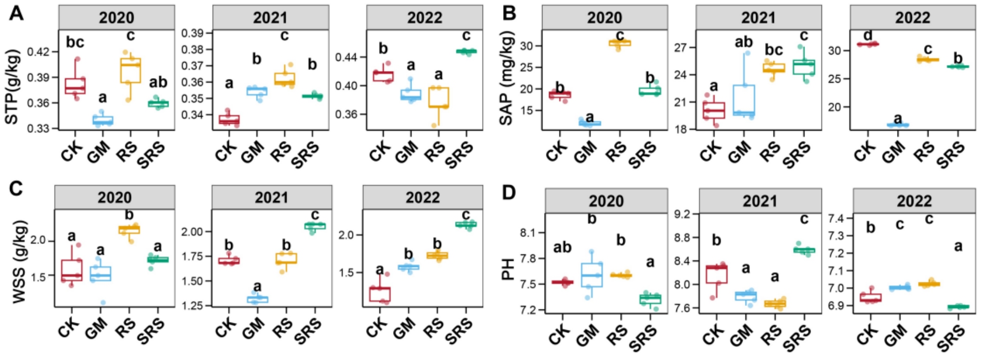

FIGURE S2 Changes in the soil total phosphorus content (A), available phosphorus content (B), water-soluble salt content (C) and pH value (D) under different treatment conditions. Different lowercase letters indicate significant differences between treatments (Tukey's test, $p < 0.05$).

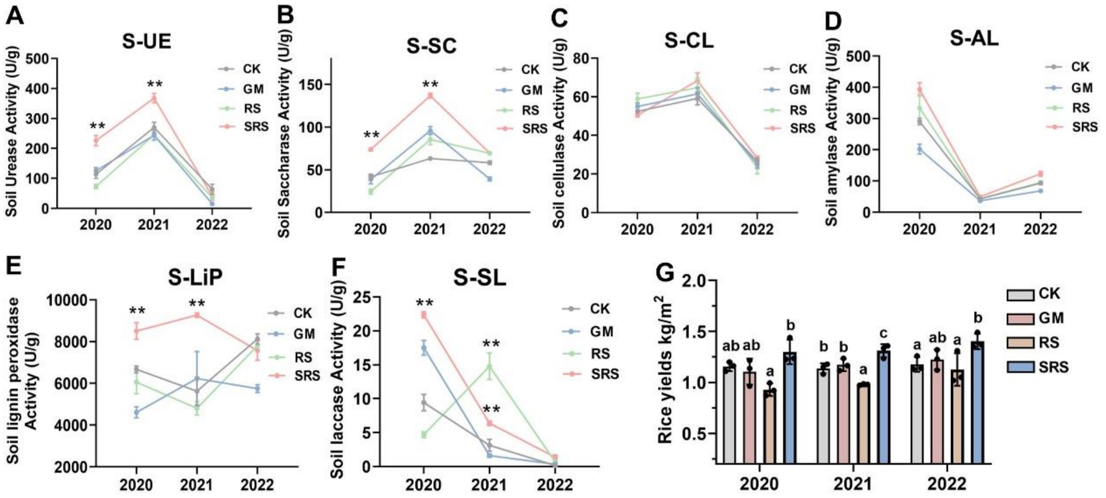

FIGURE S3 A. Soil urease (S-UE) activity; B. soil saccharase (S-SC) activity; C. soil cellulase (S-CL) activity; D. soil amylase (S-AL) activity; E. soil lignin peroxidase (S-Lip) activity; F. soil laccase (S-SL) activity; G. rice yields with different treatments. Error bars represent the standard deviation of the mean. Bars with different letters are significantly different at $P < 0.05$ according to Tukey's test, and "**" represents a significant difference at the level of $P < 0.01$.

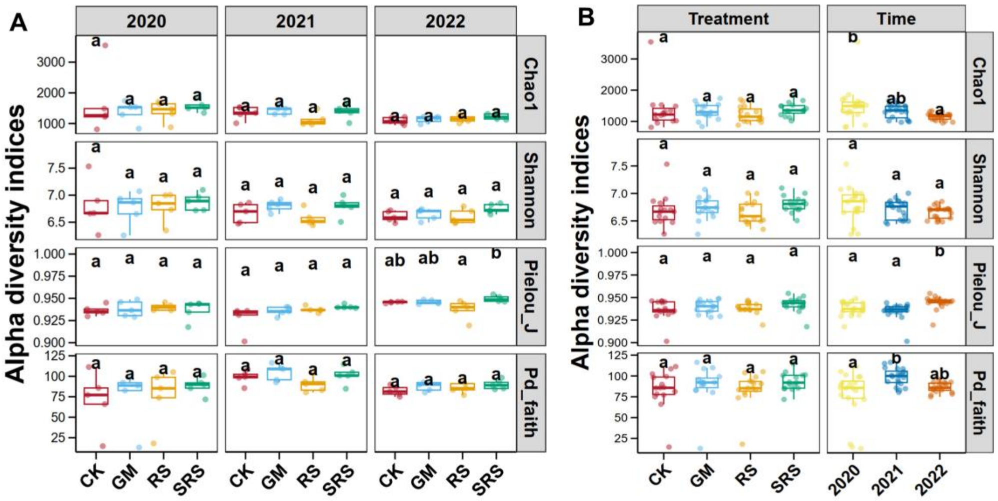

FIGURE S4 Changes in the alpha diversity index of samples of bacteria in different treatments in the same year (A) and at the interannual level (B). Different lowercase letters indicate significant differences between treatments (Tukey's test, $P < 0.05$).

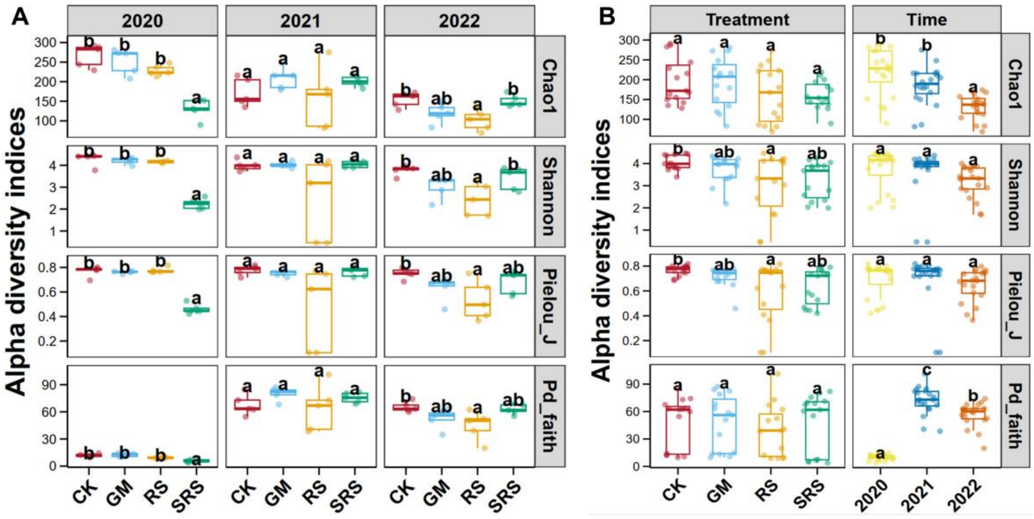

FIGURE S5 Changes in the alpha diversity indices of fungal samples from different treatments in the same year (A) and at the interannual level (B). Different lowercase letters indicate significant differences between treatments (Tukey's test, $P < 0.05$).

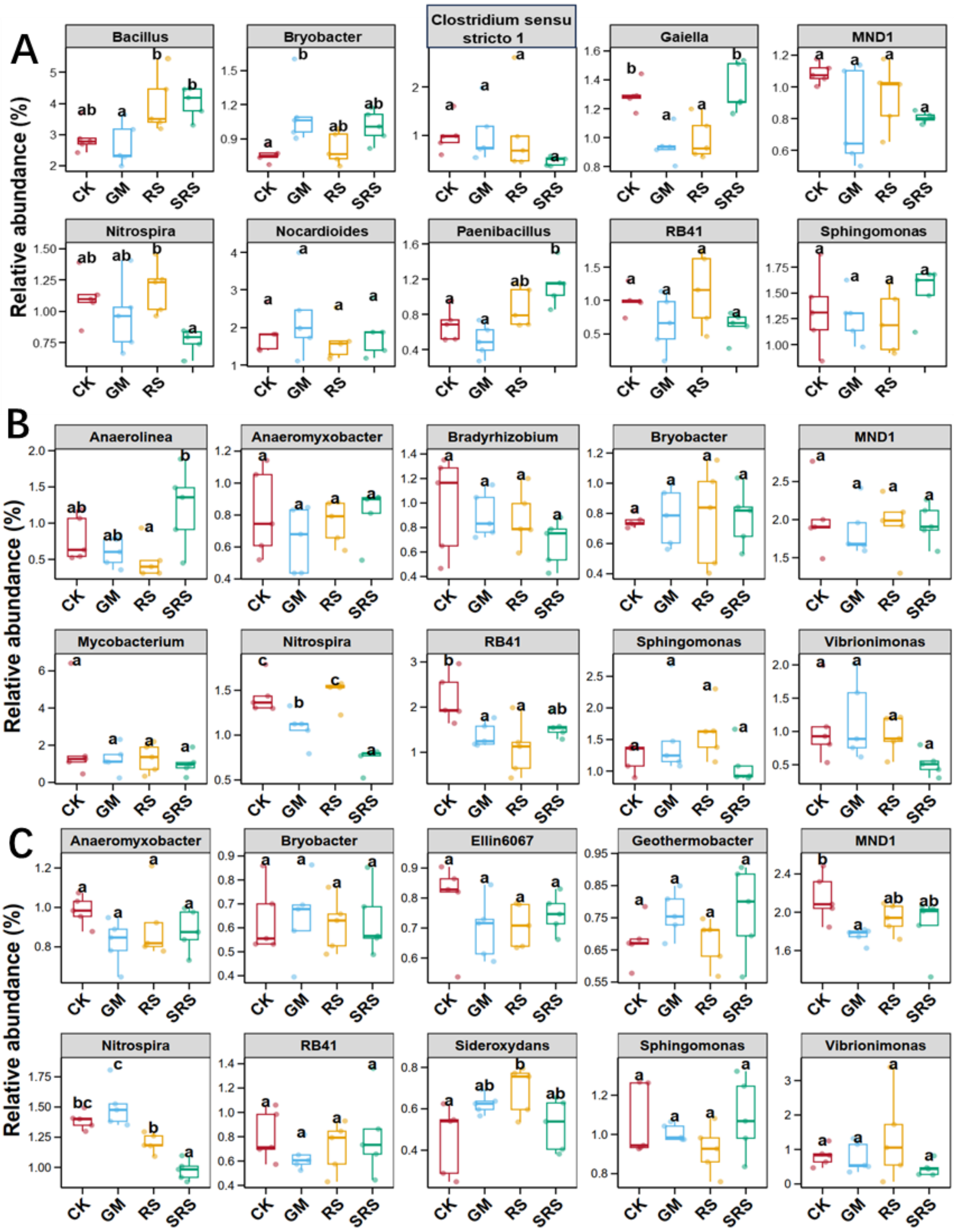

FIGURE S6 The top 10 species with large changes in soil bacterial abundance under different treatments in 2020 (A), 2021 (B) and 2022 (C). Different lowercase letters indicate significant differences between treatments (Tukey's test, $P < 0.05$).

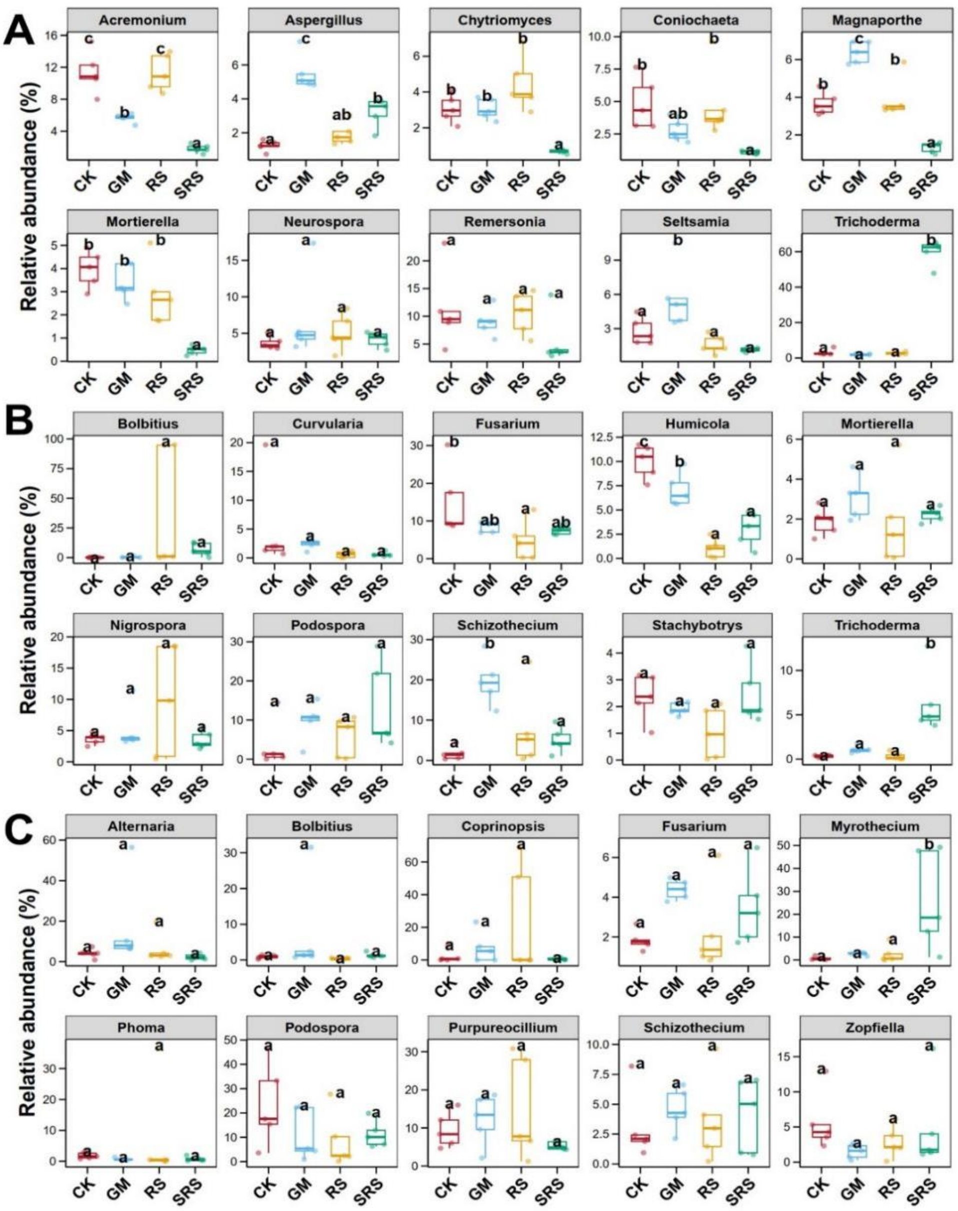

FIGURE S7 The top 10 species with large changes in soil fungal abundance under different treatments in 2020 (A), 2021 (B) and 2022 (C). Different lowercase letters indicate significant differences between treatments (Tukey's test, $P < 0.05$).

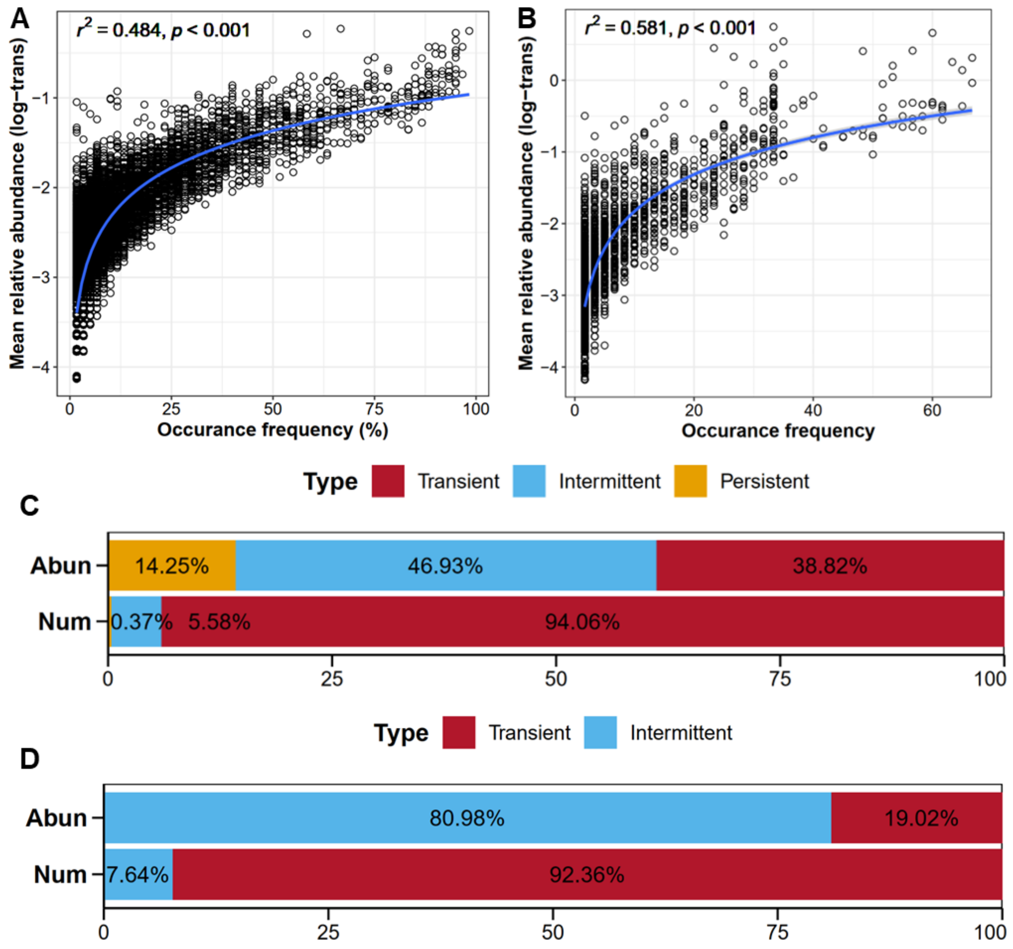

FIGURE S8 The detection rate of bacteria (A) and fungi (B) in relation to their mean relative abundance and proportion of bacteria (C) and fungi (D) classified by persistent species, intermediate species and transient species.

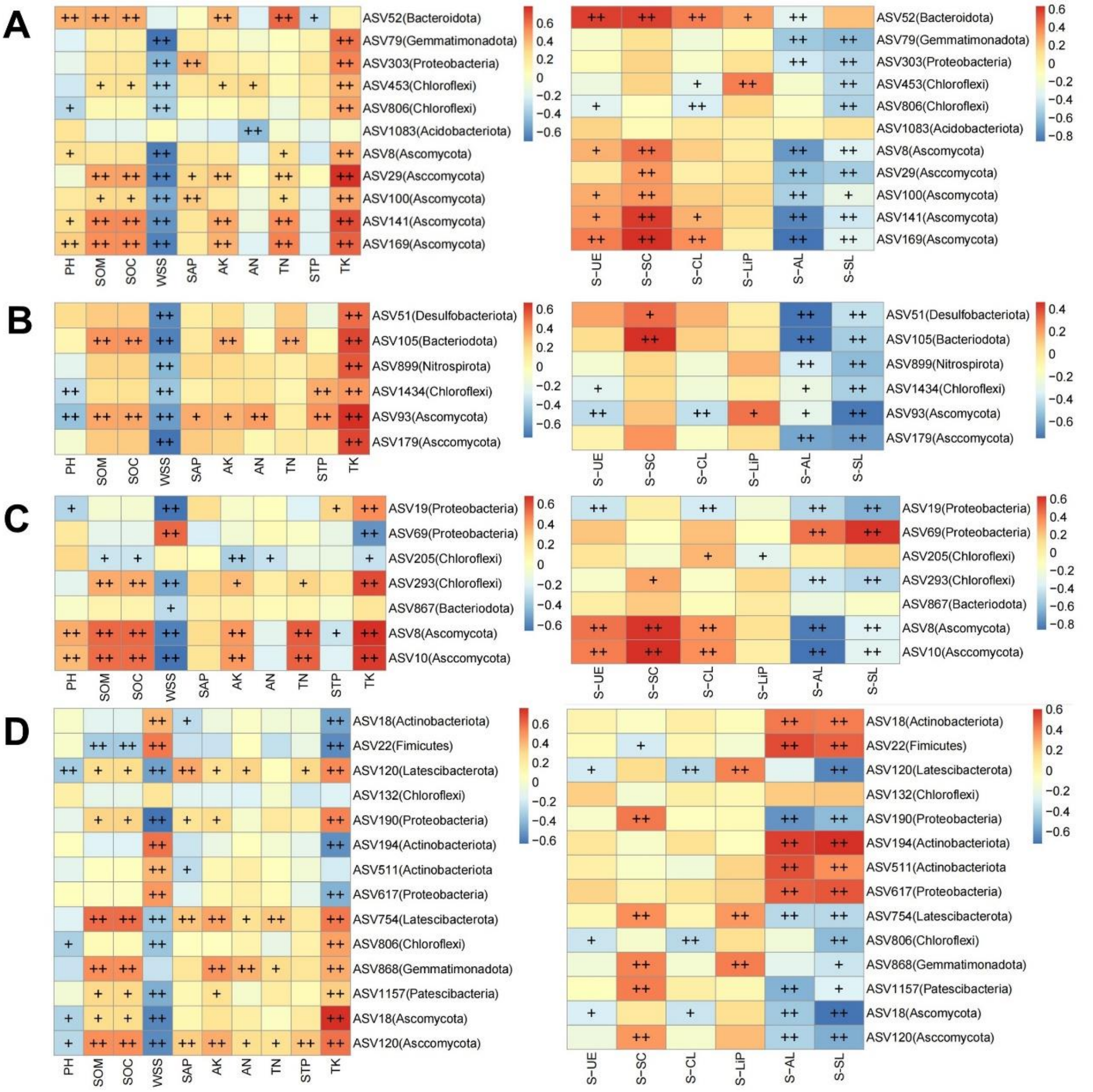

FIGURE S9 Spearman correlation analysis of key species and nutrient and enzyme activities in paddy soil. SOM: soil organic matter; SOC: soil organic carbon; TN: total nitrogen; AN: alkaline hydrolyzable nitrogen; TK: total potassium; AK: available potassium; STP: soil total phosphorus; SAP: soil available phosphorus; WSS: water-soluble salt; S-UE: soil urease; S-SC: soil saccharase; S-CL: soil cellulase; S-AL: soil amylase; S-Lip: soil lignin peroxidase; S-SL: soil laccase.
